# Theoretical and Experimental Bacterial Adherence on Chitosan Films with Varied Characteristics

**DOI:** 10.3390/ijms27104202

**Published:** 2026-05-08

**Authors:** Anouar Mouhoub, Amine Guendouz, Zainab El Alaoui-Talibi, Saad Ibnsouda Koraichi, Cherkaoui El Modafar

**Affiliations:** 1 Centre d’Agrobiotechnologie et Bioingénierie, Unité de Recherche Labellisée CNRST (Centre AgroBiotech, URL-CNRST 05), Faculté des Sciences et Techniques, Université Cadi Ayyad, Marrakech 40000, Morocco; 2 Laboratoire de Biotechnologie Microbienne et Molécules Bioactives, Faculté des Sciences et Techniques, Université Sidi Mohamed Ben Abdellah, Fès 30500, Morocco

**Keywords:** bacterial adhesion, biodegradable film, chitosan, contact angle, degree of deacetylation, zeta potential

## Abstract

This paper highlights the importance of chitosan’s intrinsic parameters on its performance as a film. Fungal (FC) and crustacean (CSC) chitosans with similar molecular weight (400 kDa) and different deacetylation degrees (FC DDA = 84.2%; CSC DDA ≈ 75%) were utilized to elaborate eco-friendly and functional chitosan-based films (C-films). The physicochemical properties as well as bioactivities were evaluated. Results showed that DDA was positively correlated with the zeta potential of the film-forming solutions. Furthermore, the FC films showed a decrease in moisture and swelling levels by about 20%, accompanied by a slight drop in qualitative hydrophobicity. On the other hand, the antibacterial activity of FC film was significantly stronger against Gram-negative bacteria compared to CSC film. Additionally, the C-films considerably mitigated the adherence of *Staphylococcus aureus*, *Pseudomonas aeruginosa*, and *Escherichia coli*, where the percentage of the covered surface ranged from 0.5 to 24%. Conversely, *Enterococcus faecalis* was more resistant, with percentages of the covered surface higher than 50%. Nonetheless, disintegration in cell structure was noticed regarding the CSC film. Ultimately, the theoretical prediction of cell adherence was highly correlated with experimental results (r = −0.89). These promising results demonstrate that C-films with high DDA are excellent candidates for preventing biofilm formation.

## 1. Introduction

Biofilm development on abiotic surfaces is presumed to be one of the main concerns in healthcare and food sectors, considering the frequency of the phenomenon and the resistance of the formed microbial structure [1,2,3]. In the food industry, microbial biofilms are considered a continuous source of contamination, compromising safety standards and reducing the shelf life of the product [1]. Similarly, in clinical settings, the development of biofilms on medical devices such as implants, catheters, and prostheses can cause problematic infections that require device removal and/or prolonged antibiotic treatments [1].

It was claimed that 40 to 80% of bacterial cells have the aptitude to switch from planktonic to sessile lifestyle and thereby contribute to the establishment of biofilms [4]. Firstly, planktonic cells approach the surface, then a reversible attachment, driven mainly by weak interactions, occurs. At this stage, the microbial cells can still detach easily [5]. During irreversible adhesion, flagella gene expression is reduced, and biofilm matrix constituents are produced, which strengthen both cell–cell and cell–surface interactions and enhance drug tolerance [5]. As biofilm matures, cell clusters appear (maturation I stage) before progressing into fully developed microcolonies (maturation II stage) [5]. Eventually, the dispersion stage is associated with the reduction and degradation of matrix constituents, during which released cells regain motility and show increased susceptibility to antimicrobial agents compared to biofilm-associated cells [5]. This phenomenon enables the colonization of new environments and ensures the persistence of the biofilm lifecycle.

Among bacteria, *S. aureus*, *S. viridans*, *P. aeruginosa*, *E. faecalis*, *E. coli*, *P. mirabilis*, *K. pneumonia*, and *S. epidermidis* are the most common species to develop microbial biofilms [6]. In this form, microorganisms benefit from multiple advantages, e.g., protection against antimicrobial agents and environmental stresses [7], due to plasmid transfer and the production of extracellular polymeric substances [8,9]. The latter presents about 80% of the biofilm’s total volume, and it is composed mainly of polysaccharides, lipids, extracellular DNA, proteins, and metal ions [10], ensuring the adsorption of the required nutrients as well as the irreversible attachment of cells to the support [11,12]. Thus, it is recommended to prevent the initial step of attachment where the microorganism–support bonding is considered weak, and adhesion is therefore reversible [13]. In other words, once established, the eradication of microbial biofilms becomes significantly complicated. Besides their intrinsic resistance, the highly organized three-dimensional structure of microbial biofilms allows cells to function as coordinated systems through quorum-sensing mechanisms, which refers to the cell-to-cell communication in the microbial community [14]. In fact, the quorum sensing regulates gene expression involved in resistance, adhesion, and virulence [15].

In this context, several techniques are used, such as biocides, ions, and antibiotic coatings of biomedical devices [16]. Nevertheless, these approaches may alter the chemical properties of the material [17]. Another procedure that involves steel coating using plastic is applied in the food industry [18]. However, plastic-based materials result in environmental pollution. On the other hand, the long-term application of conventional antimicrobial procedures may trigger the emergence of resistant strains [19]. These limitations have driven the attention of the scientific community toward the development of sustainable alternatives that combine environmental compatibility with antibiofilm efficiency. Such strategies include the utilization of eco-friendly and functional materials that derive from polymers [20,21]. Nevertheless, achieving a balance between environmental sustainability, antimicrobial efficacy, cost effectiveness, and material stability remains a considerable challenge. Multiple advanced materials involve expensive additives or require a complex process of fabrication, which limits their application on an industrial scale [22]. Thus, there is a growing interest in scalable and simple solutions that are integrated into existing medical and industrial systems.

Chitosan (C) is one of the most utilized biopolymers in this regard due to its availability in nature, biological activities, and biocompatibility [23]. Furthermore, this biopolymer presents a unique chemical structure, allowing its ability to form nanoparticles, hydrogels, coatings, and films, which makes it suitable for a wide range of applications, notably drug delivery, food packaging, water treatment, and wound healing [24,25,26,27].

In the context of an antiadhesive strategy, C-film is an excellent candidate that can act as both an active antiadhesive surface and a physical barrier. Nonetheless, C-films exhibit weaker bioactivities compared to soluble chitosan due to the regression in the chains’ positive charge [28]. This decrease leads to a mitigation in electrostatic interactions with the bacterial membrane, which are crucial for antimicrobial potency. Additionally, other factors such as surface morphology, hydrophobicity, porosity, and crystallinity can significantly affect the antibiofilm activity of the material [29,30,31]. To overcome that, previous researchers suggest the enrichment of the C-films with natural and bioactive molecules, including plant extracts and essential oils [32,33], and despite the encouraging results, the high cost of the material as well as the release of the bioactive compounds are considered a disadvantage.

For this reason, we aimed to assess the effect of chitosan deacetylation degree (DDA) on the general performance of the material, especially in terms of inhibiting the bacterial adherence of some nosocomial and food-related bacteria, and subsequently emphasize the potential of non-enriched C-films by only modifying the intrinsic parameters of this polymer, notably the DDA. This feature directly affects the density of the amino groups, and accordingly, the overall charge of the biopolymer [34]. Changes in this parameter can lead to considerable variations in viscosity, intermolecular interactions, and solubility, all of which impact C-film formation and performance [35,36,37].

In this context, FC and CSC chitosan with similar Mw and different DDA were utilized. To the best of our knowledge, the combined investigation of theoretical and experimental adhesion of biofilm-forming bacteria on C-films with varying DDAs has not been previously reported, making this study an original contribution to the field. By combining these perspectives, it becomes possible to correlate molecular-level interactions to macroscopic observations, thus providing a better understanding.

In addition, the utilization of bacterial strains relevant to both food and healthcare industries highlights the practical significance of this work. The investigation of these microorganisms’ behavior on C-film surfaces can provide valuable information regarding the potential application of this material under practical operating conditions, where diverse environmental conditions and microbial communities are encountered.

## 2. Results

### 2.1. Determination of the Zeta Potential

**Figure 1:**
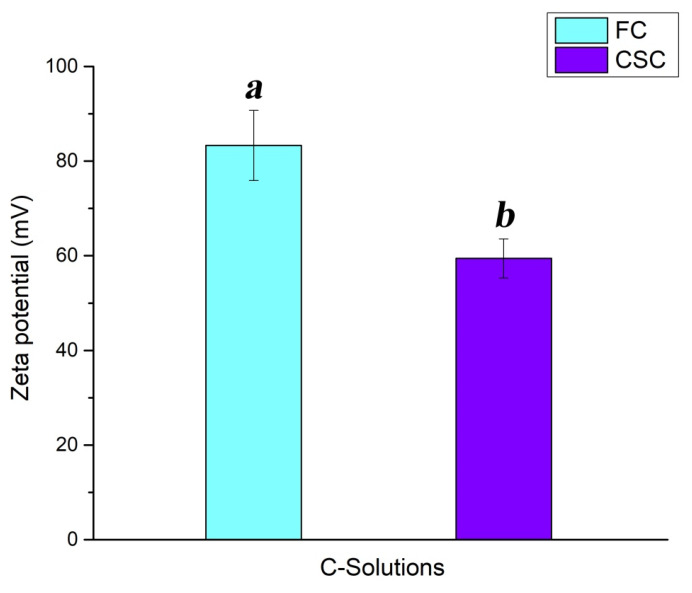
Zeta potential of FC and CSC film-forming solutions. ^a,b^ refer to zeta potential groups.

Figure 1 illustrates the difference in zeta potential between the FC and CSC film-forming solutions. Results show a significant difference between the two solutions (*p* < 0.05), with the highest value in the case of the FC solution (FC solution ≈ 83 mV; CSC solution ≈ 60 mV).

### 2.2. Evaluation of the C-Film Moisture Content, Swelling Level, and Hydrosolubility

Table 1 summarizes the C-film/water interaction. Based on the obtained findings, water constitutes about half of the C-films’ fresh weight, with values ranging between 41% and 51%. Moreover, the FC film exhibits lower moisture and swelling levels compared to the CSC film (*p* < 0.05). Nevertheless, no significant difference was noticed between the C-films as regards the hydrosolubility that exceeds 60% (*p* < 0.05).

### 2.3. Evaluation of the Physicochemical Features Concerning Bacterial Lawns and C-Films

**Figure 2:**
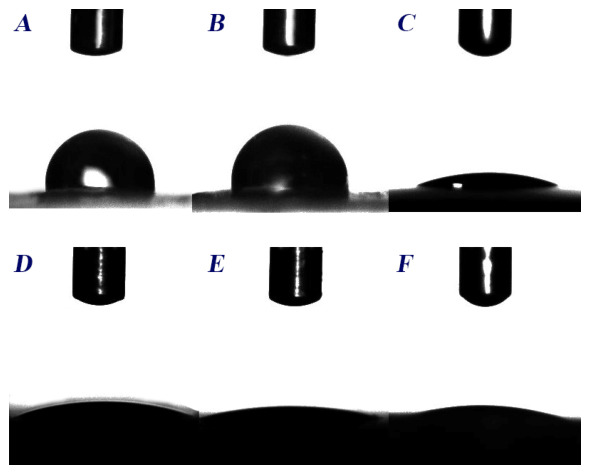
Water contact angle on the surface of (**A**) CSC film, (**B**) FC film, (**C**) *E. coli*, (**D**) *S. aureus*, (**E**) *E. faecalis*, and (**F**) *P. aeruginosa* lawns.

Table 2 presents the physicochemical characteristics of both C-films and bacterial lawns. According to the obtained data, the prepared C-films show high values of water contact angle (θ*_w_* > 65°) and negative ΔG*_iwi_* values (−6.79 and −7.20), which indicate their hydrophobic behavior (Figure 2). Dissimilar findings were noticed in the case of the microbial lawns (θ*_w_* < 65° and ΔG*_iwi_* > 0). Results also revealed that γ*^LW^* and γ^−^ forces are the most involved at the surfaces of C-films and bacterial lawns, respectively. However, both materials and lawns were essentially electron donors (γ^−^ > γ^+^).

### 2.4. Assessment of the Antibacterial Potency of the C-Films

**Figure 3:**
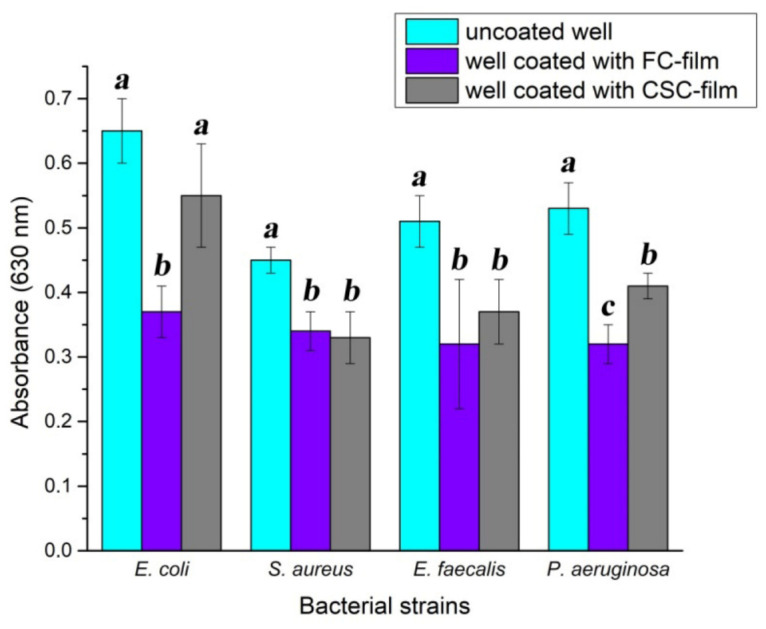
Antibacterial potency of C-films, determined by turbidity. ^a,b,c^ refer to the absorbance groups for the same strain.

As shown in Figure 3, C-films exerted a significant inhibitory effect on bacterial growth (*p* < 0.05). For Gram-negative strains, this inhibition was stronger in the case of the FC film. Nevertheless, no significant difference was noticed between the C-films activities regarding Gram-positive bacteria (*p* < 0.05). Except for the CSC–*E. coli* case, C-films inhibited the growth of all tested strains to a similar extent, resulting in turbidity values around approximately 0.35 at 630 nm, compared with values exceeding 0.45 in the untreated control.

### 2.5. Theoretical Adherence

The ΔG^Total^ values describing the theoretical adhesion of bacterial cells on C-films are presented in Table 3 and Table 4. Negative ΔG^Total^ values were observed for all tested strains except *P. aeruginosa*, indicating the favorable adherence of the three other strains to C-films. Theoretically, *E. faecalis* adherence is the most favored for both materials, followed by *E. coli*, *S. aureus*, and then *P. aeruginosa*.

### 2.6. Experimental Adherence

**Figure 4:**
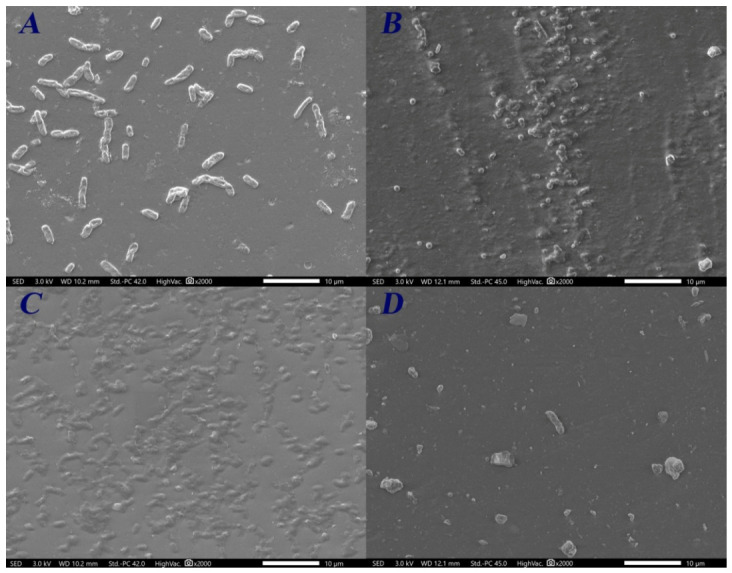
Adherence of (**A**) *E. coli*, (**B**) *S. aureus*, (**C**) *E. faecalis*, and (**D**) *P. aeruginosa* cells on FC film after 10 h of incubation.

**Figure 5:**
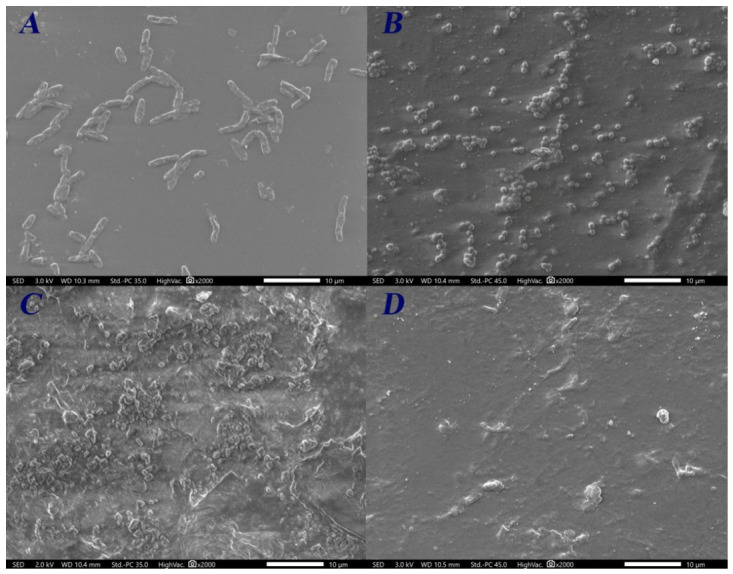
Adherence of (**A**) *E. coli*, (**B**) *S. aureus*, (**C**) *E. faecalis*, and (**D**) *P. aeruginosa* cells on CSC film after 10 h of incubation.

**Figure 6:**
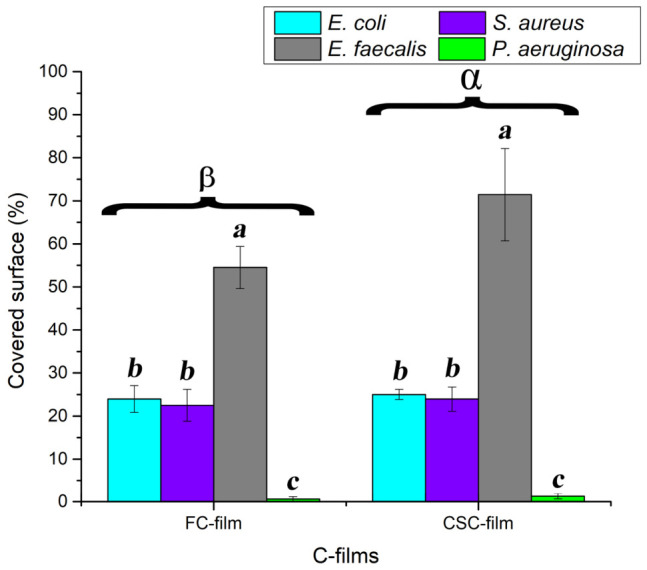
Percentage of the C-films surface covered by the bacterial cells. ^a,b,c^ refer to bacteria groups for the same C-film, while ^α,β^ correspond to C-film groups.

The cells’ adherence to FC and CSC films after 10 h of incubation is presented in Figure 4 and Figure 5, respectively. Micrographs revealed different cell affinities for C-films. For both C-films, *E. faecalis* cells exhibited the highest percentages of adherence, with values of material-covered surface higher than 50% (Figure 6). On the other hand, the adherence of *P. aeruginosa* cells was minimal (covered surface < 2%). Furthermore, SEM micrographs concerning the Gram-positive bacteria show the initiation of the biofilm establishment. Nevertheless, the structure of *E. faecalis* cells on CSC film seems to be disrupted.

### 2.7. Correlation Between the Theoretical Adhesion and Experimental Results

**Figure 7:**
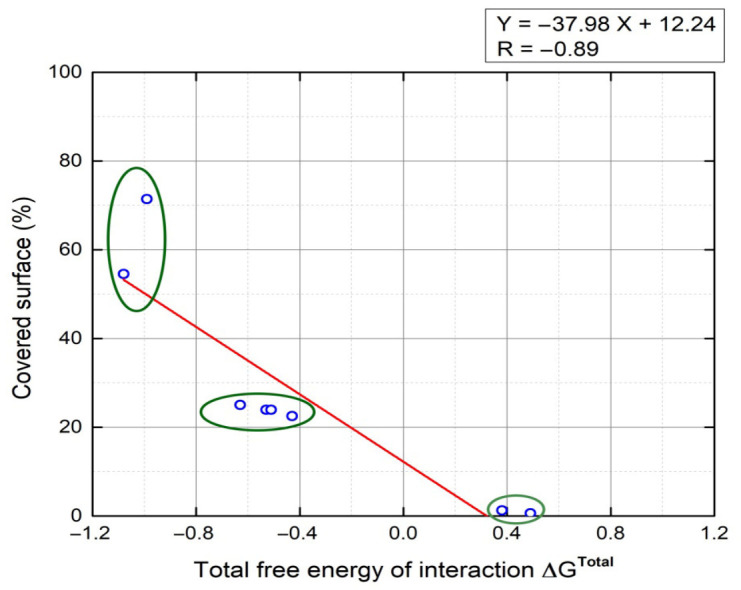
Correlation between the prediction of bacterial cell adherence and experimental results.

Figure 7 illustrates the correlation between ΔG*^Total^* and bacterial adherence. The theoretical and experimental adhesion data showed a strong inverse correlation (r = −0.89), reflecting a consistent relationship between both datasets. Based on the results, three groups of covered surface percentages were noticed: the first (covered surface > 50%) when ΔG*^Total^* ≤ −1, the second (covered surface ≈ 25%) when −0.8 ≤ ΔG*^Total^* ≤ −0.4, and the third (covered surface ≈ 0%) when 0 < ΔG*^Total^*.

## 3. Discussion

The DDA is one of the two factors that determine the properties and biological activities of chitosan [38]. Results revealed a positive correlation between the DDA and zeta potential of the C-solutions. In fact, higher DDA signifies more exposed amino groups, resulting in a higher positive charge. The latter could be responsible for the decrease in moisture and swelling levels observed in the case of the FC film. Basically, the swelling process of materials occurs when H_2_O molecules interact with the hydrophilic groups of the material-forming molecules. Hence, an increase in free amino groups is expected to engender a rise in the swelling level. Nevertheless, the intermolecular bonds formed between the hydroxyl and amino groups can restrict water from accessing the crystalline regions of the film [39]. Results also showed that the prepared C-films exhibit considerable hydrosolubility (around 60%). Several studies have highlighted the water sensitivity of chitosan-based films [40,41]. This could be related to the partial protonation of the amino groups in aqueous conditions, leading to a potential repulsion between the cationic chains [42]. Due to the dissolved carbon dioxide, the distilled water presents an acidic pH (pH < 6), which is responsible for the formation of the NH_3_^+^ groups. These chemical groups are also linked to the antimicrobial potency of chitosan. It was previously reported that chitosan inhibits microorganism growth through multiple mechanisms of action [43,44]. The well-known suggestion implicates the interaction between the positively charged biopolymer and the negatively charged components located on the surface of the microorganism cells [45,46], which causes the permeabilization of the cell membrane and then the leakage of intracellular content. This confirms the antibacterial activity engendered by the soluble fraction of the C-films against the four tested strains. Multiple investigations have reported the antimicrobial activity of chitosan-based solutions [47]. In general, a concentration around 1 g/L is considered efficient [48].

The observed variability in C-film activity could be explained by the disparity in chitosan DDA and in the cell wall composition [43]. In the case of Gram-positive bacteria, the anionic teichoic acids present in the peptidoglycan layer can non-covalently bind to the chitosan molecule [49]. These phosphorus-containing polymers are vital for growth, enzyme activity, resistance to rough environments, and cell division [50]. Additionally, a recently published paper demonstrated the involvement of teichoic acids in *S. aureus* adhesion to surfaces and biofilm establishment [51]. Meanwhile, cationic amino groups of chitosan interact with phosphoryl and carbonyl groups of the phospholipids that constitute the membrane of Gram-negative bacteria [52]. Another work claims the association of chitosan with nucleic acids of microorganisms, resulting in the inhibition of mRNA and protein synthesis [53].

Besides the direct bactericidal action, the ability to inhibit microbial adherence is a required property for antimicrobial materials. Generally, this characteristic is influenced by the physicochemical features of both material and microorganism [54]. According to the results, the bacterial cells showed a hydrophilic behavior. It is well known that, except for extremophiles, bacterial cells are generally hydrophilic [55]. On the other hand, the two C-films were qualitatively and quantitatively hydrophobic. However, the FC film showed a slight reduction in the water contact angle, which might be explained by the higher proportion of the free amino groups. While numerous studies have emphasized the hydrophilic nature of chitosan [56,57], the observed hydrophobicity of C-films could be due to several factors including the presence of the N-acetyl group [58], the binding between anionic OH^−^ groups of glycerol and cationic NH_3_^+^ groups of chitosan leading to the exposition of the glycerol hydrophobic region [40,59], and the contamination of the film surface by hydrophobic monomers especially during the drying process [60]. A number of authors have stated that hydrophilic surfaces are more suitable for hydrophilic cell adherence [61,62]. Moreover, the negative charge of the bacterial surface, attributed to phospholipids and teichoic acid for Gram-negative and Gram-positive bacteria, respectively, promotes the attachment to materials with the opposite charge [63]. Accordingly, the bacterial adherence must be favored on the prepared C-films. Nevertheless, this phenomenon depends on other factors, including the non-specific Lifshitz–Van der Waals interactions and the specific attachment mediated by adhesins [64], which explains the variability in the percentage of colonization between the four tested strains. Additionally, the Gram-positive strains seem to develop a biofilm structure faster than the Gram-negative ones. The latter are known to use acyl-homoserine lactone-mediated quorum sensing to regulate the biofilm establishment, while the Gram-positive bacteria use autoinducing peptides [65]. Finally, a disruption of *E. faecalis* structure was observed. This could be related to the interaction between the soluble fraction of the CSC film and the bacterial cells, leading to membrane disintegration and thereby death of cells. Consequently, longer incubation may reveal a clearer inhibition of the developed microcolonies.

## 4. Materials and Methods

### 4.1. Materials

Fungal (FC) and crustacean C3646 (CSC) chitosans were acquired from BioLaffort (BioLaffort, Floirac, France) and Sigma-Aldrich (Sigma–Aldrich, St. Louis, MI, USA), respectively. The biopolymers’ characteristics are listed in Table 5.

The tested strains were *S. aureus* (CIP 543154, Collection of Institut Pasteur, Paris, France), *P. aeruginosa* (ATCC 27653, American Type Culture Collection, Manassas, VA, USA), *E. faecalis* (ATCC 29212, American Type Culture Collection, Manassas, VA, USA), and *E. coli* (CIP 5412, Collection of Institut Pasteur, Paris, France).

The utilized chemicals, solvents, and media were potassium nitrate (Sigma-Aldrich, Schnelldorf, Germany), acetic acid (VWR, Fontenay-sous Bois, France), methylene iodide (Sigma-Aldrich, Schnelldorf, Germany), formamide (Sigma-Aldrich, Schnelldorf, Germany), glycerol (Solvachim, Casablanca, Morocco), Lysogeny broth (Labkem, Barcelona, Spain), and Mueller Hinton Agar (Biokar diagnostics, Beauvais, France).

### 4.2. Preparation of the Microbial Suspensions

For the antibacterial test, the suspensions were prepared by the inoculation of Lysogeny broth with fresh cultures of the tested strains. As regards the antiadhesion test, the bacterial cells were rinsed twice using sterile potassium nitrate solution (0.1 M) and then dispersed in the same solvent [67]. For both tests, the suspensions were adjusted to an optical density of 0.4 at 600 nm, corresponding to an approximate cell density of 10^8^ cells/mL under the experimental conditions [68].

### 4.3. Preparation of the Cell Lawns

The bacterial suspensions prepared in potassium nitrate solution were filtered using negative pressure and then dried at room temperature for 1 h to obtain the cells’ lawns.

### 4.4. Preparation of the C-Films

The C-films were prepared by dissolving C-powder (2% *w*/*v*) in 1% acetic acid solution. Afterward, glycerol at 750 μL per 1 g of C-powder was added to the C-solution under continuous stirring. The obtained mixture was evaluated in terms of zeta potential and then dried overnight on glass supports at 30 °C. Finally, the C-films have been detached from the supports and then stored for subsequent analyses.

### 4.5. Zeta Potential Determination

The zeta potential of FC and CSC solutions was determined using a Zetasizer apparatus (Nano ZS Malvern Instruments, Worcestershire, UK). The assessment was performed at 25 °C with a minimum of 12 runs. Ultrapure water with a conductivity of about 0.055 µS/cm was used to rinse the Zetasizer cell before each use to ensure measurement stability.

### 4.6. C-Films’ Interaction with Water

The moisture (M%) and swelling (S%) percentages, as well as the hydrosolubility (HS) of the C-films, were defined by measuring the weight after the soaking and drying processes as mentioned in our previous study [69].

### 4.7. Physicochemical Features of the Bacterial Lawns and C-Films

The physicochemical features of the bacterial lawns and C-films’ surfaces were defined as suggested in our prior work [70]. Concisely, a GBX tensiometer (GBX Instruments, France) was used to calculate the contact angles of three fluids with known characteristics (H_2_O, CH_2_I_2,_ and CH_3_NO). The surface free energy (γ_S_^Total^) and its components, as well as the free energy of interaction (ΔG*iwi*), were defined by the following equations.

$$\gamma_{F}\left(1+\mathrm{Cos}\theta \right)={2\left({\gamma_{F}^{\mathrm{LW}}\gamma }_{S}^{\mathrm{LW}}\right)}^{1/2}{+{2\left({\gamma_{F}^{+}\gamma }_{S}^{-}\right)}^{1/2}+2\left({\gamma_{F}^{-}\gamma }_{S}^{+}\right)}^{1/2}$$

$$\gamma_{S}^{\mathrm{Total}}=\gamma_{S}^{\mathrm{AB}}+\gamma_{S}^{\mathrm{LW}}\;\mathrm{where}\;\gamma_{S}^{\mathrm{AB}}={2\left({\gamma_{S}^{+}\gamma }_{S}^{-}\right)}^{1/2}$$

$$\Delta \mathrm{Giwi}=-2[2({{\left(\gamma_{w}^{-}\gamma_{w}^{+}\right)}^{1/2}+(\gamma_{i}^{+}\gamma_{i}^{-})}^{1/2}-{\left(\gamma_{i}^{+}\gamma_{w}^{-}\right)}^{1/2}-{\left({\gamma_{w}^{+}\gamma }_{i}^{-}\right)}^{1/2})+({\left(\gamma_{i}^{\mathrm{LW}}\right)}^{1/2}-{{\left(\gamma_{w}^{\mathrm{LW}}\right)}^{1/2})}^{2}]$$

where θ: Contact angle;

F: Fluid phases;
S: Solid surface;
γ^−^: Lewis base;
γ^+^: Lewis acid;
γ*^LW^:* Lifshitz–Van der Waals component.

The surface hydrophobicity can be predicted quantitatively and qualitatively based on ΔG*iwi* and water contact angle (*θ*_w_), respectively. Hydrophobic surfaces are characterized by a negative ΔG*iwi* value and *θ*_w_ value higher than 65°, and conversely for hydrophilic surfaces [71,72].

### 4.8. Antibacterial Activities of the C-Films

Firstly, the microplate wells were covered with FC and CSC solutions, oven-dried, and then UV sterilized. Following drying, the formation of a uniform, thin, and transparent film on the well surface was visually observed, indicating consistent surface coverage. Untreated microplate wells without any polymer layer (uncoated) served as controls. Next, 0.2 mL of the suspension was added and incubated overnight at 30 °C. The antibacterial potency of the C-films was concluded by the calculation of turbidity at 630 nm using a Jenway 6305 UV-Visible spectrophotometer (Jenway, Stone, Staffordshire, UK). The tests were performed in triplicate.

### 4.9. Theoretical Adhesion

Different from the DLVO theory, the developed XDLVO theory includes polar interaction. Considering the high ionic strength of the potassium nitrate utilized in this study as a suspending solution, electrical interactions were neglected [73]. Thus, the total free energy of interaction (${\Delta G}_{\mathrm{MSW}}^{\mathrm{Total}}$) between microbial cell (M) and substrate (S) via water (W) is determined as follows:

$${\Delta G}_{\mathrm{MSW}}^{\mathrm{Total}}={{\Delta G}_{\mathrm{MSW}}^{\mathrm{LW}}+\;\Delta G}_{\mathrm{MSW}}^{\mathrm{AB}}$$

where ${\Delta G}_{\mathrm{MSW}}^{\mathrm{LW}}$ represents the contribution to the free energy of interaction between the microbial surface (M) and the solid surface (S) in water (W) arising from Lifshitz–van der Waals forces.

$${\Delta G}_{\mathrm{MSW}}^{\mathrm{LW}}=({\left(\gamma_{S}^{\mathrm{LW}}\right)}^{1/2}{{\left(\gamma_{M}^{\mathrm{LW}}\right)}^{1/2})}^{2}-{((\gamma_{M}^{\mathrm{LW}})}^{1/2}-{{\left(\gamma_{W}^{\mathrm{LW}}\right)}^{1/2})}^{2}-{((\gamma_{S}^{\mathrm{LW}})}^{1/2}-{{\left(\gamma_{W}^{\mathrm{LW}}\right)}^{1/2})}^{2}$$

And ${\Delta G}_{\mathrm{MSW}}^{\mathrm{AB}}$ represents the contribution to the free energy of interaction between the microbial surface (M) and the solid surface (S) in water (W) arising from acid–base interactions.

$${\Delta G}_{\mathrm{MSW}}^{\mathrm{AB}}=2[{\left(\gamma_{W}^{+}\right)}^{1/2}({\left(\gamma_{M}^{-}\right)}^{1/2}+{\left(\gamma_{S}^{-}\right)}^{1/2}-{\left(\gamma_{W}^{-}\right)}^{1/2})+{\left(\gamma_{W}^{-}\right)}^{1/2}({\left(\gamma_{M}^{+}\right)}^{1/2}+{\left(\gamma_{S}^{+}\right)}^{1/2}-{\left(\gamma_{W}^{+}\right)}^{1/2})-{\left({\gamma_{S}^{-}\gamma }_{W}^{+}\right)}^{1/2}-{\left({\gamma_{S}^{+}\gamma }_{W}^{-}\right)}^{1/2}]$$

Bacterial cell adherence is favored when ${\Delta G}_{\mathrm{MSW}}^{\mathrm{Total}}$ presents a negative value [74].

### 4.10. Experimental Adhesion

Samples of FC and CSC films (1 cm × cm) were placed in beakers containing the KNO_3_–bacterial suspensions and then incubated at 30 °C for 10 h, corresponding to the early stage of bacterial adherence and biofilm formation. Next, the C-films were washed using sterile H_2_O to eliminate the non-adherent bacterial cells. Three different areas of each sample were scanned using a scanning electron microscope (JEOL, JSM-IT500 HR, Tokyo, Japan). The SEM micrographs were processed using Photoshop Version 13.0 x32 and MATLAB software R2016b (version 9.1) to determine the percentage of cell adherence [67].

### 4.11. Statistical Analysis

Findings were displayed as mean ± SD. The significant differences were defined via the ANOVA test using the SPSS software v 25.0. The significance level was set at 5%.

## 5. Conclusions

In summary, the variation in DDA impacted C-film characteristics such as the moisture and swelling levels and the physicochemical features. The latter were correlated with the antiadhesion action of the material. Moreover, the film-forming solution prepared from the FC chitosan showed a higher zeta potential (≈83 mV), confirming the strong antibacterial effect of the FC film against two of the tested bacteria, notably *E. coli* and *P. aeruginosa,* compared to the CSC film. Furthermore, the antiadhesive potency of the FC film was slightly better than that of the CSC film against *E. faecalis*. However, CSC film engendered structure disruption with regard to the adhered cells. This insight reveals that the optimization of surface charge can lead to the enhancement of the C-film antimicrobial activity.

In general, findings emphasize the important role of intrinsic biopolymer features in controlling the global performance of the elaborated C-film, particularly in the context of antimicrobial activity. By proving that changes in DDA alone can significantly impact both the physicochemical characteristics and biological activities, this work demonstrates that effective antiadhesive materials can be prepared without the need for additional incorporation of bioactive compounds or chemical modification.

Ultimately, future investigation should also explore the mechanical and thermal properties, long-term stability, and biodegradability of these films under conditions that simulate application in the packaging industry, as well as their efficiency against more complex and multispecies biofilms. Tests using chitosan with DDA > 95% are recommended as well in order to investigate whether chitosan with a higher DDA further enhances the biological activity of the developed material. Such investigations would further validate the practical potential of DDA-optimized chitosan films as sustainable antiadhesive packaging and coating.

## Figures and Tables

**Table 1:** Values of moisture and swelling percentages, as well as hydrosolubility.

	M (%)	S (%)	HS (%)
FC film	41.19 ± 3.02 ^b^	628.31 ± 30.04 ^b^	63.09 ± 1.95 ^a^
CSC film	50.89 ± 2.57 ^a^	751.08 ± 20.08 ^a^	61.30 ± 2.09 ^b^

^a,b^ refer to the moisture content, swelling level and hydrosolubility groups.

**Table 2:** Physicochemical features of the C-films and bacterial lawns.

C-Films and Bacterial Lawns	Contact Angles (°)			Surface Free Energy Components (mJ/m^2^)					ΔGiwi (mJ/m^2^)
	θ CH_2_I_2_	θ CH_3_NO	θ H_2_O	γ*^LW^*	γ^+^	γ^−^	γ*^AB^*	γ*^Total^*	
FC film	39.27 ± 0.15 ^c^	98.57 ± 1.08 ^b^	90.73 ± 0.46 ^b^	39.89	12.00	22.84	33.11	73.00	−7.20
CSC film	51.73 ± 0.55 ^a^	104.23 ± 0.68 ^a^	96.07 ± 0.21 ^a^	33.23	10.77	19.66	29.10	62.33	−6.79
*E. coli*	29.57 ± 1.04 ^e^	29.17 ± 0.67 ^c^	17.13 ± 0.15 ^c^	44.30	0.04	60.93	3.12	47.42	45.53
*S. aureus*	29.43 ± 0.55 ^e^	28.37 ± 0.21 ^c^	15.57 ± 0.67 ^d^	44.36	0.04	61.43	3.13	47.49	45.97
*E. faecalis*	32.23 ± 0.45 ^d^	12.20 ± 0.92 ^d^	10.53 ± 0.67 ^e^	43.81	0.71	55.18	12.52	56.33	32.71
*P. aeruginosa*	42.47 ± 0.80 ^b^	15.43 ± 2.55 ^d^	10.93 ± 0.29 ^e^	38.27	1.33	55.75	17.22	55.49	33.02

^a–e^ refer to the contact angle groups for the same solvent.

**Table 3:** Total free energy of interaction ΔG*^Total^* between the bacterial cells and the FC film.

FC Film–Bacterial Cell Interaction	ΔG*^LW^* (mJ/m^2^)	ΔG*^AB^* (mJ/m^2^)	ΔG*^Total^* (mJ/m^2^)
*E. coli*	−6.70	6.19	−0.51
*S. aureus*	−6.72	6.29	−0.43
*E. faecalis*	−6.42	5.34	−1.08
*P. aeruginosa*	−5.13	5.62	0.49

**Table 4:** Total free energy of interaction ΔG*^Total^* between the bacterial cells and the CSC film.

FC Film-Bacterial Cells Interaction	ΔG*^LW^* (mJ/m^2^)	ΔG*^AB^* (mJ/m^2^)	ΔG*^Total^* (mJ/m^2^)
*E. coli*	−4.49	3.86	−0.63
*S. aureus*	−4.50	3.97	−0.53
*E. faecalis*	−4.30	3.31	−0.99
*P. aeruginosa*	−3.44	3.82	0.38

**Table 5:** Characteristics of the utilized chitosans.

Chitosans	Source	Deacetylation Degree (DDA)	Molecular Weight (Mw)
FC	Fungi	84.2% [66]	400 kDa [66]
CSC	Shrimp	≈75%	400 kDa

## Data Availability

The datasets presented in this article are not readily available because the data are part of an ongoing study. Requests to access the datasets should be directed to the corresponding author.

## Abbreviations

The following abbreviations are used in this manuscript:

FC	Fungal chitosan
CSC	Crustacean chitosan
C-film	Chitosan-based film
DDA	Deacetylation degree
Mw	Molecular weight
M%	Moisture content
S%	Swelling degree
HS	Hydrosolubility
